# Feasibility outcomes of a presurgical randomized controlled trial exploring the impact of caloric restriction and increased physical activity versus a wait-list control on tumor characteristics and circulating biomarkers in men electing prostatectomy for prostate cancer

**DOI:** 10.1186/s12885-016-2075-x

**Published:** 2016-02-05

**Authors:** Wendy Demark-Wahnefried, Jeffery W. Nix, Gary R. Hunter, Soroush Rais-Bahrami, Renee A. Desmond, Balu Chacko, Casey D. Morrow, Maria Azrad, Andrew D. Frugé, Yuko Tsuruta, Travis Ptacek, Scott A. Tully, Roanne Segal, William E. Grizzle

**Affiliations:** Department of Nutrition Sciences, University of Alabama at Birmingham (UAB), 346 Webb Nutrition Sciences Bldg., 1675 University Blvd, Birmingham, AL USA; Department of Urology, UAB, Birmingham, AL USA; Department of Human Studies, UAB, Birmingham, AL USA; Department of Preventive Medicine, UAB, Birmingham, AL USA; Department of Molecular & Cellular Pathology, UAB, Birmingham, AL USA; Department of Cell, Developmental & Integrative Biology, UAB, Birmingham, AL USA; Department of Microbiology, UAB, Birmingham, AL USA; Urology Centers of Alabama, Birmingham, AL USA; Department of Medicine, University of Ottawa, Ontario, Canada; Department of Pathology, UAB, Birmingham, AL USA

**Keywords:** Prostatic neoplasms, Diet, Physical activity, Exercise, Weight loss, Obesity, Intervention, Presurgical

## Abstract

**Background:**

Obesity is associated with tumor aggressiveness and disease-specific mortality for more than 15 defined malignancies, including prostate cancer. Preclinical studies suggest that weight loss from caloric restriction and increased physical activity may suppress hormonal, energy-sensing, and inflammatory factors that drive neoplastic progression; however, exact mechanisms are yet to be determined, and experiments in humans are limited.

**Methods:**

We conducted a randomized controlled trial among 40 overweight or obese, newly-diagnosed prostate cancer patients who elected prostatectomy to explore feasibility of a presurgical weight loss intervention that promoted a weight loss of roughly one kg. week^−1^ via caloric restriction and physical activity, as well as to assess effects on tumor biology and circulating biomarkers. Measures of feasibility (accrual, retention, adherence, and safety) were primary endpoints. Exploratory aims were directed at the intervention’s effect on tumor proliferation (Ki-67) and other tumor markers (activated caspase-3, insulin and androgen receptors, VEGF, TNFβ, NFκB, and 4E-BP1), circulating biomarkers (PSA, insulin, glucose, VEGF, TNFβ, leptin, SHBG, and testosterone), lymphocytic gene expression of corresponding factors and cellular bioenergetics in neutrophils, and effects on the gut microbiome. Consenting patients were randomized in a 1:1 ratio to either: 1) weight loss via a healthful, guidelines-based diet and exercise regimen; or 2) a wait-list control. While biological testing is currently ongoing, this paper details our methods and feasibility outcomes.

**Results:**

The accrual target was met after screening 101 cases (enrollment rate: 39.6 %). Other outcomes included a retention rate of 85 %, excellent adherence (95 %), and no serious reported adverse events. No significant differences by age, race, or weight status were noted between enrollees vs. non-enrollees. The most common reasons for non-participation were “too busy” (30 %), medical exclusions (21 %), and “distance” (16 %).

**Conclusions:**

Presurgical trials offer a means to study the impact of diet and exercise interventions directly on tumor tissue, and other host factors that are feasible and safe, though modifications are needed to conduct trials within an abbreviated period of time and via distance medicine-based approaches. Pre-surgical trials are critical to elucidate the impact of lifestyle interventions on specific mechanisms that mediate carcinogenesis and which can be used subsequently as therapeutic targets.

**Trial registration:**

NCT01886677

## Background

Obesity is increasingly recognized as a risk factor for cancer [1]. Currently, there is consensus that obesity serves as a risk factor for eight different malignancies, i.e., endometrial, colorectal, renal, esophageal, breast (post-menopausal), thyroid, gall bladder, and pancreas [2–4]. Moreover, obesity also serves as a poor prognostic indicator for several other cancers – at least 15 in total [5]. In prostate cancer, obesity is not associated with the overall risk for disease, but it does place men at increased risk for more aggressive cancer and disease-specific mortality [6]. A recent multinational study involving 10,106 prostate cancer cases from eight cohorts with an average follow-up of 8.2 years found that each 5 unit increase in prediagnostic body mass index (BMI: kg/m^2^) was associated with an 8 % increase in mortality (p-trend = 0.01) [7]. Weight gain after diagnosis and primary treatment was examined in an earlier study among 26,479 prostate cancer patients; here, each 5 unit increase in BMI was associated with 21 % increased risk of biochemical recurrence (Relative Risk: 1.21, 95 % Confidence Interval: 1.11-1.31 P < 0.01) [8].

Despite strong observational evidence that a higher BMI is associated with more aggressive and progressive cancer, major gaps exist in our understanding of that relationship with key research questions being: Are weight loss interventions feasible in populations with cancer? Does intentional weight loss result in improved cancer control? What are the mechanisms by which negative energy balance affects tumor biology and the host environment? Are the effects of caloric restriction and increased energy expenditure through physical activity similar or do they differ?

To date, there have been roughly 20 weight loss trials among various oncology patient populations that have been completed or are currently in the field that address some of these questions. Most of these trials have been conducted in breast cancer survivors and are modest in size; results show feasibility, safety, and a significant impact on reducing adiposity and improving health-related quality of life - largely focusing on physical functioning and fitness [9]. In addition, many have assessed the impact of weight loss on circulating biomarkers, such as insulin and related entities (insulin-like growth factors and binding proteins), adipokines, inflammatory markers, sex steroid hormones, and related binding proteins. Findings have been compiled in a review by Reeves et al. [9] and show significant reductions in insulin in 2-of-6 studies [10–15], and leptin in 3-of-3 studies [10, 14, 15]; however, other results are inconclusive largely due to inadequate statistical power. As of yet, no studies have been completed that assess the impact of intentional weight loss on recurrence or cancer-specific mortality, though there are currently two European trials in the field with this goal, i.e., the Simultaneous Study of Docetaxel-Gemcitabine Combination adjuvant treatment, and Extended Bisphosphonate and Surveillance (SUCCESS-C) and the Diet and Androgens (DIANA-5) trials [16, 17]. In prostate cancer, there have been only three reported weight loss studies. The largest of these, the RENEW trial (Reach Out to ENhancE Wellness in Older Cancer Survivors) enrolled 261 prostate cancer survivors within a study cohort that also included 380 other survivors of breast and colorectal cancer [18]. In this study, significant reductions in body weight occurred and were associated with improvements in physical functioning (the primary endpoint of the trial). The two other randomized trials have been modest in size with sample sizes of 8 and 19 [19, 20], and also showed successful weight loss. In the trial by Wright et al. [20] pre-post changes in serum insulin-like growth factor binding protein (IGFBP)-3 were observed between the control (−6.9 %) and intervention groups (+2.8 %); though no differences were observed in insulin, c-peptide, IGF-1 and adiponectin – again, likely due to inadequate power. Despite the fact that both of these last two trials were conducted in the presurgical setting, neither investigated the impact of caloric restriction on tumor tissue.

The ability to ascertain the impact of interventions directly on tumor tissue is considered a particular strength of presurgical trials, and it is reasoned that by monitoring intervention effects on Ki-67 proliferation rates (a well-accepted tumor marker used for pharmacologic studies and also one that has shown to be sensitive to changes in diet and nutritional status) [21–23], the efficacy of an intervention could be assessed in a much shorter period of time and with much smaller numbers of participants; moreover, the biological mechanisms through which the intervention exerts its therapeutic potential could be ascertained directly. Originally proposed as a resourceful way of testing chemopreventive agents, Kelloff and colleagues proposed the use of presurgical models over a wide range of cancers in a hallmark paper published over two decades ago [24]. Since this time, they have been used for the evaluation of therapeutic agents [22, 25–28], but have been used far less frequently to assess the impact of complementary therapies that encompass diet and exercise with the expressed intent of assessing the impact of the intervention on the tumor. While the biological effects of lifestyle interventions are believed to be far less potent than pharmacological agents, a phase II RCT conducted among 161 patients scheduled for prostatectomy found significantly lower Ki-67 proliferation indexes in men randomized to receive a 3-week regimen of 30 g/day of ground flaxseed vs. those who did not receive it [21, 29]. Thus, this trial serves as proof of concept that presurgical trials are indeed viable and valuable for testing lifestyle interventions. However, their use has not been evaluated in studies of energy balance that are aimed at assessing the impact of caloric restriction or increased physical activity on tumor tissue.

The purpose of this paper is to describe a pioneering NIH-funded (R21 CA161263) RCT that utilizes a pre-surgical model to explore the feasibility and effects of a diet and exercise weight loss intervention on tumor proliferation rates (Ki-67), as well as other outcomes in men with newly-diagnosed prostate cancer. Herein, we describe the study design, research protocol, and the necessary adjustments made in order to conduct the presurgical weight loss trial in men who elected prostatectomy as their first line of treatment for prostate cancer.

## Methods

### Design/Specific aims

**Fig. 1:**
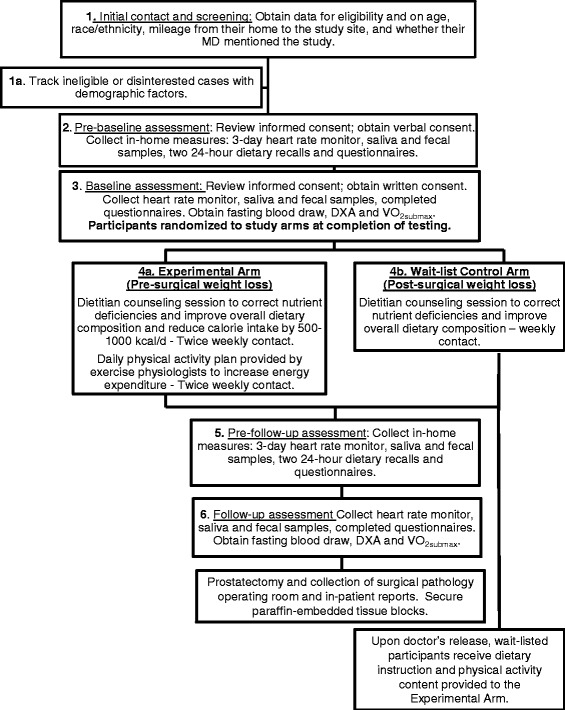
Study schema

This two-armed (experimental arm: assigned immediately to a healthful energy-restricted diet + exercise intervention to promote a mean weight loss of 2 pounds/week vs. wait-list control arm: assigned to receive the intervention post-surgery) randomized controlled trial (RCT) among 40 overweight or obese men newly-diagnosed with prostate cancer was designed as a feasibility study to explore whether weight loss during the presurgical period was feasible and associated with favorable changes in tumor biology and the host environment. While the basic structure of the trial is almost identical to that of the original NIH application, some modifications were necessary to overcome barriers and to implement the trial in a real-world setting; these modifications are indicated in the subsequent paragraphs. This protocol and all amendments were approved by the Institutional Review Board of the University of Alabama at Birmingham (F11051002), in compliance with the Helsinki Declaration, and registered and reported according to Consolidated Standards of Reporting Trials guidelines (NCT01886677). Fig. 1 provides an illustration of the study schema, and specific aims are listed below (note that both the feasibility and exploratory aims were fully established at the time of grant submission and remained unaltered):

- Feasibility aims
  To determine the feasibility of the trial against common process and safety benchmarks, i.e., accrual (completion of targeted enrollment [*n* = 40] within two years) and retention (≥80 %) in the overall sample, adherence (completion ≥70 % of contact sessions), and safety (absence of serious, life-threatening adverse events) in the experimental intervention arm.
  To quantify (mean change scores and variance) and compare differences in body weight over the study period between the experimental vs. the wait-list control arms.
- Exploratory aims
  To obtain means and precision estimates and explore pre-post between-arm differences on the following: 1) other adiposity measures (waist circumference [WC], and % body fat); 2) energy intake and physical activity (minutes/week and metabolic equivalents [METs]); 3) serum markers and lymphocytic gene expression related to insulin, leptin, testosterone, sex hormone binding globulin (SHBG), Tumor Necrosis Factor (TNF)-β, Vascular Endothelial Growth Factor (VEGF), phosphatidylinositol 3 kinase (PI3K), and Phosphatase and tensin homolog (PTEN) and prostate specific antigen (PSA), and 4) functional and health-related outcomes, e.g., V0_2submax_ and quality of life (QoL).
  To obtain means and precision estimates and explore between-arm differences with regard to select clinical outcomes (margin positivity, surgical complications, operating time, and length of hospital stay) and also the following tumor markers: Ki-67, activated caspase-3 (apoptosis), VEGF, androgen and insulin receptors (AR and IR), PTEN, nuclear factor kappa-light-chain-enhancer of activated B cells (NFκB), and serine/threonine kinase (AKT).
  To conduct correlative science exploration to assess associations between 1) measures of adiposity and energy intake and expenditure with biomarkers in the sera, lymphocytes, and prostate tissue; 2) Ki-67 with other markers within the tumor; and 3) levels of circulating biomarkers compared with those in the tumor.
- Additional aims
  Two collaborative studies were added onto the original NIH-funded investigation, and were supported by institutional funding. The first is aimed at evaluating of the effect of the weight loss intervention on the mitochondria. This study, led by BC, is exploring the impact of negative energy balance on cellular bioenergetics and the respiratory burst in neutrophils and monocytes, and also is exploring associations between mitochondrial bioenergetic profiles and potential mediators and biomarkers of prostate cancer progression, such as rates of proliferation and apoptosis in the tumor tissue and serum PSA. The second is aimed at exploring the potential effects of the weight loss intervention and any specific effects of caloric restriction or increased physical activity on the gut microbiome (CDM and TP).

### Eligibility/Accrual

Prostate cancer patients were recruited from the urology clinics at the University of Alabama at Birmingham (UAB) and also the Urology Centers of Alabama. In most cases, patients were initially contacted by telephone, the study was explained and interest in the study was elicited, along with data on race/ethnicity, age, and distance from the study site. Patients who were not interested were thanked for their time and then asked to volunteer their reasons for disinterest; this information was de-identified and recorded. Patients who expressed an interest were then screened for eligibility using the eligibility criteria listed in Table 1. Ineligible patients’ data also were de-identified and recorded in the database.

**Table 1:** Inclusion and exclusion criteria

Inclusion criteria	Exclusion criteria
• Adult men (age 19+) with pathologically confirmed prostate cancer who elected prostatectomy as their primary initial treatment. • Scheduled for prostatectomy at one of the participating study sites. • Potential lag-time between baseline (which had to be scheduled at least 2 weeks from prostatic biopsy) and follow-up appointments of at least 23 days ^a^. • Overweight or obese, but not class III morbidly obese (BMI range 25 to ≤50 kg/m^2^). • Telephone access.	• Previous hormonal or neo-adjuvant chemotherapy. • Another active malignancy (exception non-melanoma skin cancer) in addition to prostate cancer. • Current health or medical condition that affects weight status, e.g., untreated hyper- or hypo-thyroidism, etc. • Pre-existing medical condition that precludes adherence to unsupervised exercise, e.g., severe orthopedic conditions, scheduled for a hip or knee replacement, boney metastases, paralysis, dementia, untreated stage 3 hypertension, or unstable angina, heart attack, congestive heart failure or conditions that dictated hospitalization or oxygen within 6-months. • Unable to read or speak English • Unwillingness to be randomized to either the immediate weight loss intervention or the wait-listed control arms. • Enrolled in a formal weight loss program.

^a^ This criteria was changed from that of the original study (i.e., 10 weeks), because potential recruits were unwilling to delay their surgery; therefore, we modified this criteria to correspond to that of our previous RCT in which we found significant effects with a 3-week dietary regimen. It should be noted despite this reduction in the criteria, the mean length of time that participants were on study was 7 weeks^21,29^

Written consent from men who were eligible and interested in participating was obtained using two different procedures depending on the convenience of the patient and the distance that they resided from the study site. For the first option, consent was obtained during a pre-baseline appointment at which a 24-h hour dietary recall was conducted and men were provided with a programmed heart rate monitor (to collect 3 days of physical activity data), fecal and saliva specimen home collection containers, and questionnaires to complete on sociodemographic factors, comorbidity, and QoL. For the second option, men were express mailed the consent form and all of the study materials mentioned previously, and a 24-h dietary recall was conducted over the telephone after obtaining verbal consent; written consent was then obtained at the baseline assessment.

Baseline assessments were scheduled at the convenience of the patient and to accommodate a 12-h fast. Vital signs were taken and any participants exhibiting uncontrolled Stage III hypertension (>99 diastolic or >159 systolic at rest) or cardiac abnormality were cleared by their urologist prior to randomization [30]; one patient required clearance and would have been omitted from the study had they not been cleared, since there have been serious cardiovascular events in previous exercise studies among prostate cancer patients) [31]. At both this visit and at follow-up (scheduled to accommodate a 12-h fast and within 3-days of prostatectomy) testing was performed to assess anthropometrics, body composition via dual energy x-ray absorptiometry (DXA), fitness using VO_2submax,_ and to collect blood (see section on Measures/Measurement Points for detail). To help defray costs associated with study participation and reduce potential barriers to accrual, men were offered a monetary incentive of $15 for each clinic assessment they completed, as well as $15/week to participate in clinic-based physical activity sessions.

### Randomization

After all baseline data and biospecimens were collected, men were randomized stratified on race (African American vs. others) and baseline BMI (25–29.9 vs. 30+) to assure an even balance amongst study arms regarding both factors which have been found previously to influence disease progression or intervention adherence [32, 33]. Men were assigned to one of two study arms: (1) the Experimental Arm that received the weight loss regimen immediately; or 2) the Wait-List Control Arm that was offered the weight loss regimen after completion of the study period. The randomization sequence and group allocation were generated by the biostatistician (RAD) using a computer-generated random number sequence (PROC PLAN SAS® Ver. 9.4). Sealed envelopes with study assignments were created and numbered; envelopes within each strata were opened in sequence and assignments were verified against the original sequence patterns.

### Interventions

#### Experimental arm (immediate weight loss regimen)

Participants in this arm received counseling from a registered dietitian (WDW, MA) on a healthy, nutritionally-adequate diet which met the Dietary Reference Intakes as well as food choice patterns consistent with guidelines of the American Cancer Society (ACS) and the World Cancer Research Fund – American Institute of Cancer Research [34, 35]. The Mifflin-St. Jeor equation with an assumption of sedentary behavior [(10 × body weight in kg) + (6.25 × height in cm) – (5 × age) × 1.2], [36] was used to obtain energy needs; subtraction of 1000 kcal/day was then used to promote an average weight loss of two pounds/week [36]. Participants were provided with references and instructed to count their calories or use the “Choose Your Foods” American Diabetes and Dietetic Association exchange list system [37]. Food group distribution was customized to suit patients’ needs and preferences; dietitians consulted 24-h dietary recalls to alert participants of suboptimal nutrient intakes in an effort to correct any nutritional inadequacies through food sources. Participants were instructed to count their calories each day, as well as their minutes of exercise. “The Calorie King: Calorie, Fat and Carbohydrate Counter” (Family Health Publications, Costa Mesa, CA) was provided along with a pocket-size log book. Participants were instructed to weigh themselves daily and provided with a scale if they did not have one [38]. Dietitian follow-up occurred twice weekly with counseling either provided face-to-face, via the telephone or through email. In each session, dietary intakes were reviewed, reinforcement provided, and challenges of the upcoming week were discussed in an effort to problem solve and arrive at potential strategies. The overarching theoretical framework for this intervention was provided by Social Cognitive Theory which emphasizes the importance of self-efficacy, skills development, and self-monitoring in producing behavior change [39].

In addition, exercise physiologists also provided counseling on regimens that ultimately would afford up to an additional 250 to 500 kcal (kcal)/day deficit primarily through aerobic exercise. As per the American College of Sports Medicine (ACSM) guidelines, each training session included not only the work-out, but also a 5-min warm-up and cool down of slow walking and stretching [40]. An incremental approach for the workout was employed with ramping of intensity and volume from 60 to 80 % of maximum heart rate (MHR) as tolerance permitted. Participants were encouraged to exercise at UAB twice weekly under supervision where they trained on ergometers and treadmills, and 5 times/week at home; they were provided with heart rate monitors and instruction for use during these sessions to enhance and monitor adherence. Trainers employed similar scheduling and approaches to that of the dietitians and downloaded heart rate monitors regularly. Given that some participants resided hundreds of miles from the study site, on-site training was not possible and necessitated a regimen that relied exclusively on regular telephone counseling and/or email exchanges and routinely scheduled downloads of heart rate monitor data. Additional coaching was provided during each contact with the participant, regardless of whether the contact was made by the dietitian or the exercise trainer, since we sought to use every opportunity to reinforce the intervention. The concession to deliver the weight loss regimen through a distance-medicine approach was made when we discovered that most patients who elected to receive their prostatectomies at the two study sites came from a broad catchment area that included Alabama, Arkansas, Florida, Georgia, and Mississippi.

#### Wait-listed control arm (delayed weight loss regimen)

As in the experimental arm, these participants were apprised of suboptimal nutrient intakes that were apparent from 24-h recalls and counseled by dietitians on food sources to correct any nutritional inadequacies with follow-up on a weekly basis. They also were offered the weight loss regimen with up to six weeks of follow-up once they had recovered from their surgery.

### Safety

Study participants were monitored at least weekly over the course of the study period, to discern adverse events. We were especially interested in any serious or life-threatening event (defined as a physical or cardiac event which resulted in overnight hospitalization) that was attributable to the intervention; however, none occurred.

### Measures/Measurement points

**Fig. 2:**
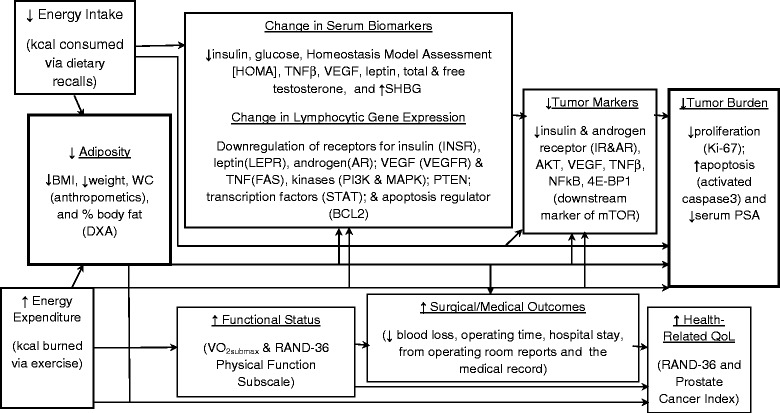
Logic model

A logic model of the study outcomes and their inter-relationships is provided in Fig. 2. With the exception of the collection of tumor tissue from diagnostic biopsies and prostatectomy, study measures were performed at baseline and follow-up (which occurred within three days prior to surgical treatment). Detail on each of these measures follows:

#### Anthropometric measures

All anthropometric measures were conducted in accordance with procedures detailed in the Anthropometric Standardization Reference Manual [41]. Height was measured at baseline only using fixed stadiometer and to the nearest 0.1 cm (without shoes). Weight was measured on a calibrated platform scale to the nearest 0.1 kg (without shoes and in light clothing with pockets emptied). BMI was calculated using the following formula: kg/m^2^. WC was measured to the nearest 0.1 cm with a non-stretch, tension-controlled tape measure at the level of the umbilicus.

#### Body composition

Total body fat mass and lean mass were measured by DXA using a regularly calibrated Lunar Prodigy densitometer (GE-Lunar Corporation, Madison, WI, software version 12.3). Participants were required to wear light clothing, remove all metal objects from their body, and lie supine with arms at their sides while undergoing a total body scan.

#### Cardiorespiratory fitness

Heart rate, oxygen consumption, ventilation, perceived exertion, and the respiratory exchange ratio were monitored during VO_2submax_ assessments. Measurements were begun with the participant seated for 5 min. The treadmill testing used increasing intensity in four minute intervals, i.e., it began at 2 miles per hour (MPH) at a 0 % incline, then 2 MPH at 4 % incline, 3 MPH at 4 % incline, 4 MPH at 4 % incline, and finally 4 MPH at 8 % incline (which few participants achieved), to a goal of 80 % of MHR, as defined by ACSM criteria [40]. Upon completion of each interval, the above measures were recorded and testing was terminated once 80 % MHR was achieved. Total time on the treadmill was then recorded.

#### Patient reported data and outcomes

At baseline, patients were asked to report all of the prescribed and over-the-counter medications (including dietary supplements) that they took; this list was reviewed at the follow-up appointment and changes were noted. At both time points self-administered questionnaires assessed comorbidity, and QoL using the Older Americans Resources and Services (OARS) Comorbidity Index [42], and the RAND-36 and Prostate Cancer Index [43, 44], respectively. Given the need to collect data within a circumscribed time period, the 7-day Physical Activity Recall was modified to a 3-day collection period and administered by trained personnel at each time point [45]. Self-reported physical activity data were further supported by the collection of objective heart rate monitor data using the Polar RS400 (Polar Electro, Inc., Lake Success, NY) for each 3-day collection period. Two-day dietary recalls were conducted by registered dietitians with random recalls of one weekday and one weekend day carefully timed at baseline and follow-up to invoke standardization [46]. The NCI-developed ASA24 was used to analyze nutrient composition and provides output on mean intakes of 64 food-related components from the USDA Nutrient Database for Dietary Studies and 31 food groups from the USDA MyPyramid Equivalents Database [47]. In this project the key data identified from this program were kilocalories, macronutrients, and nutrient density.

#### Circulating biomarkers

Blood (29.7 ml) was collected by venipuncture and separated into sera, plasma, neutrophils, leukocytes, DNA and RNA (dispersed in 0.5 ml of RNAlater®). The leukocytes and neutrophils were used immediately for studies on cellular bioenergetics and respiratory burst as previously described in papers by Chacko and colleagues [48, 49] All other samples were stored at −80 °C (except the sample in the RNAlater which was stored at −20 C). All sera will be batch analyzed in duplicate. The following biomarkers will be assayed using immunofluorescence (TOSOH AIA-II analyzer, TOSOH Corporation, South San Francisco, CA): insulin; total testosterone; SHBG; and PSA. Cytokines (TNFβ and VEGF), will be run on a MSD imager (Meso Scale Diagnostics, Rockville, MD). Radioimmunoassay will performed according to manufacturer’s directions (Millipore RIA, Billerica, MA) to ascertain leptin. Glucose will be assessed using a glucose oxidase reagent as per Stanbio Sirrus (Stanbio Labs, Boerne, TX).

In tandem with these biomarkers, we also will explore gene expression of receptors for insulin (INSR), leptin (LEPR), and androgen (AR), as well as for VEGF (VEGFR) and TNF (FAS). We also will investigate gene expression with regard to PTEN, specific kinases (PI3K & MAPK), transcription factors (STAT), and regulation of apoptosis (BCL2). The total RNA will be extracted from the samples using a Trizol reagent (Invitrogen, Carlsbad, CA) and RNeasy Mini Kit (QIAGEN, Valencia, CA) according to the manufacturer’s instructions. The concentration and purity of total RNA will be determined by 2100 Bioanalyzer (Agilent Technologies, Santa Clara, CA). cDNA will be synthesized from 1 μg RNA using High Capacity cDNA Reverse Transcription Kits (life technologies, Grand Island, NY) according to the manufacturer’s instructions. cDNA will then be amplified by real-time PCR using TaqMan Universal Master Mix II (life technologies) with a CFX Connect Real-Time PCR Detection System (Bio-Rad, Hercules, CA). Primers will be obtained from TaqMan Gene Expression Assay (life technologies, Grand Island, NY). The expression levels of genes will be normalized to the expression level of the 18 s rRNA in each sample. For mRNA analysis, the calculations for determining the relative level of gene expression will be made using the cycle threshold method.

#### Tumor biomarkers

Pathologist (WEG), blinded with regard to study condition, will review clinical pathology reports and all slides for each case; he then will choose one slide and one block per case based on the presence of adequate tumor and the histological grade of tumor on the slide will be representative of the entire tumor in the specimen. Slides will then be prepared for determination of proliferation rate (Ki-67) and other tumor markers (activated caspase 3, IR, AR, VEGF, TNFβ, NFκB, 4E-BP1). Specific antibodies and dilutions will be decided upon prior to analysis.

#### Gut microbiome

At each time point, subjects collected fecal samples in order to explore if changes in physical activity and dietary intake affected the microbiome. Fecal samples were collected after a bowel movement and using a sterile wipe which was then placed in a plastic bag. Men were asked to record the dates and times of collection. If samples were collected on days that preceded the scheduled assessment, men were instructed to freeze the sample and to submit it at the time of their appointment. Microbiome analysis targeting the V4 region of the 16S rRNA gene was performed using an Illumina MiSeq as described previously [50]. File conversion and quality control also were performed as documented earlier [51]. Analyses of the microbiome samples were performed with the Quantitative Insight into Microbial Ecology (QIIME) suite, version 1.7 [52] using a QIIME wrapper called QWRAP as described previously [50, 51].

### Statistical considerations and analyses

#### Sample size and statistical power

While this study was primarily undertaken as a feasibility pilot, initial power calculations were based on weight loss data among men ages 50 years and older enrolled in our UAB weight loss programs, who achieved a mean (SD) weight loss of 12.4 (6.89) pounds over a 10-week period. A sample size of 16/arm (which assumes 20 % attrition) yields 98 % power to detect a difference in means of 10.40 (i.e., Group 1 mean, μ_1_ of −12.40/Group 2 mean, μ_2_ of −2.00) assuming a similar SD for both groups and using a 2-group, 2-sided *t*-test and α = .05.

#### Statistical comparison

Differences between the control and experimental groups at baseline were assessed by t-tests for continuous variables, and Fisher’s exact tests or Chi-square tests for categorical variables. Median baseline PSA levels were compared using the Wilcoxon test. Changes in anthropometric measures will be computed as follow-up values - baseline values. The changes will be compared between the treatment arms using a generalized linear model with the change score as the dependent variable and the treatment group as a predictor, controlling for baseline measures. A similar model will be used for the nutrition parameters assessed by the ASA 24 [47]. The serum biomarker values that are not normally distributed will be log transformed prior to analyses. The changes from baseline to follow-up will be computed on the log-transformed data and will be compared by treatment group with linear models controlling for baseline values. Associations between tumor biomarkers and adiposity measures will be examined with Pearson’s correlations coefficients and linear regression. For bioenergetic assessments, each blood sample will be analyzed with 4–6 assay replicates and data will be presented as means ± standard errors. Statistical significance will be determined using either student’s T-tests or one-way ANOVAs, followed by Tukey’s post hoc analyses with *p* < 0.05 to designate statistical significance. Given the pilot nature of this study, no adjustments will be made for multiple comparisons.

## Results

### Accrual

**Fig. 3:**
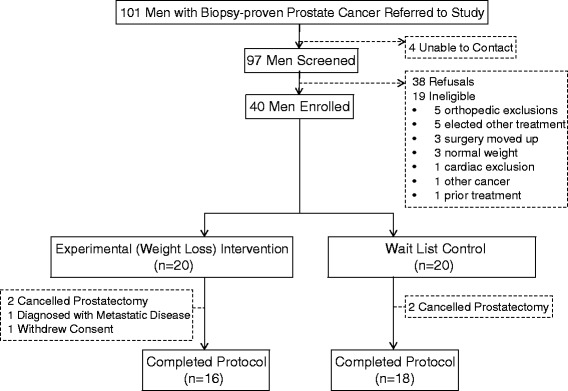
CONSORT diagram

This trial achieved all of its feasibility endpoints. Accrual was met within a 2-year period, and required the screening of 101 patients in order to enroll 40 participants (an enrollment rate of 39.6 %) (see Fig. 3 for CONSORT diagram). Given physician turnover within our center, it was necessary to partner with another institution in order to meet our target. In analyses aimed at determining differences between enrollees vs. non-enrollees, we found no differences with regard to age, race, BMI, and whether or not the patient recalled that their urologist mentioned the trial (Table 2). A non-significant trend was noted with regard to distance from the study site. Leading reasons for non-participation were “too busy” (30 %), medical exclusions (21 %), distance (16 %) and changes in plans surrounding surgery, i.e., opting for a procedure at a different site, rescheduling at an earlier date, and indecision or deciding on other forms of treatment (11 %). Eighteen percent of non-participants either expressed no reason or were unable to be contacted.

**Table 2:** Characteristics of enrollees vs. non-enrollees

Characteristic	Enrolled (*n* = 40)	Not enrolled (*n* = 61)	*P*-value
Age (years) – mean (SD)	59.9 (6.5)	61.9 (6.5)	0.13
BMI – mean (SD)	31.6 (4.4)	31.2 (4.7)	0.69
Study mentioned by physician – N (%)			
Yes	21 (52.5)	31 (50.8)	0.87
No	19 (47.5)	30 (49.2)	
Race– N (%)			
African American	12 (30.0)	15 (24.6)	0.78
White	28 (70.0)	45 (73.8)	
Other	–	1 (1.6)	
Miles from study site – N (%)			
< 50	21 (53.8)	20 (35.7)	0.08
≥ 50	18 (46.2)	36 (64.3)	

### Retention

Retention exceeded the benchmark of 80 % with 34 of the 40 participants (85 %) completing the trial. Of those who dropped-out, two-thirds did so because they decided against surgery. There were no differences between completers vs. non-completers on age, race, BMI, or Gleason score.

### Adherence

Once enrolled, participants exceeded the previously set benchmark for adherence, i.e., 95 %(9) as compared to the 70 %. However, as previously stated, the intervention was redesigned to accommodate home delivery through the provision of heart rate monitors, scales and telephone counseling.

### Safety

No adverse events were observed or reported during the intervention.

## Discussion

Only two other weight loss interventions have been pursued in the presurgical cancer setting, and neither of these small studies (*n* = 8 and *n* = 19 [of which only half were pre-surgical cases]), [19, 20] assessed effects on tumor tissue. Therefore, this trial will be the first to assess the impact of an acute period of negative energy balance directly on the biology of the tumor, as well as within the more global host environment. Given the growing interest in weight control as a complementary therapy to standard cancer treatment [1], such trials can provide the mechanistic evidence needed to justify the incorporation of diet and physical activity into oncologic practice. As such, the methods listed herein can offer a helpful framework for the design of future trials, as well as data on feasibility that can be used to specifically inform recruitment, retention, and the design of presurgical interventions.

Given the need to collect surgical specimens, presurgical trials dictate collaboration with co-investigators and cancer centers that perform substantial numbers of procedures annually. Physician turnover and changes in practice can cause unforeseen delays and in fact, occurred with this trial. Therefore, partnerships with other institutions are necessary and were instrumental in achieving targeted accrual within the two-year timeframe of this study. Nevertheless, patient indecision regarding surgical treatment and its timing pose substantial barriers to both recruitment and retention. Patients are unwilling to delay their surgery and it was clear that if we were to meet our accrual target, we needed to reduce our initial 10-week intervention period to a shorter period of time. As stated, 3-weeks was selected based on the results of our previous trial [21]. Other issues related to presurgical studies are the possibilities that surgeries can be rescheduled and patients can decide to receive their treatment elsewhere or not at all. Therefore, an adequate margin of over accrual is necessary to ultimately meet sample sizes that afford adequate power.

Our enrollment rate of almost 40 % was relatively good for a diet and exercise intervention trial among cancer survivors where reported rates of participation have ranged from 6 % to 42 % [53]. The modifications made to shorten the study period, as well as to deliver the intervention primarily through a home-based approach that relied on telephone counseling and technologic support likely enhanced our participation. Other criteria, such as those set to assure safety are not as amenable to change and the fact that we did not observe any serious adverse events provides further support to the medical exclusions that were made. However, given that distance and travel still were listed as one of the top reasons for refusal, it might be possible to overcome these barriers through an added participant incentive or a higher reimbursement rate for travel. Fifteen dollars was all the current grant could afford and may have been too low a stipend especially if the distance between the patient’s home and the study site is substantial.

Finally, both retention and adherence exceeded the benchmarks established for this trial. Moreover, with the exception of only two men who were dissatisfied with their randomization status, there was solid rapport between the study staff and participants.

## Conclusion

This presurgical feasibility trial met all benchmarks related to accrual, retention, adherence, and safety. As analyses are completed, the potential impact of the weight loss intervention on these biologic outcomes can be determined and subsequently reported. Until then, these methods have many applications that go far beyond the scope of this investigation.

## Abbreviations

7-Day PAR: seven day physical activity recall
AKT: serine/threonine kinase
AR: androgen receptor
BMI: body mass index
CV: coefficient of variation
DIANA-5: DIet ANd androgens trial
DXA: dual energy x-ray absorptiometry
HOMA: Homeostasis Model Assessment
IGF: insulin-like growth factor
IGFBP: insulin-like growth factor binding protein
IR: insulin receptor
KCAL: kilocalorie
METS: metabolic equivalents
MHR: maximal heart rate
MPH: miles per hour
NFκB: nuclear factor kappa-light-chain-enhancer of activated B cells
OARS: Older Americans Resources and Services
PI3K: phosphatidylinositol 3 kinase
PTEN: phosphatase and tensin homolog
PSA: prostate specific antigen
QoL: quality of life
RCT: randomized controlled trial
RENEW: Reach out to ENhancE wellness in older cancer survivors trial
SD: standard deviation
SE: standard error
SHBG: sex hormone binding globulin
TNF: tumor necrosis factor
UAB: University of Alabama at Brimingham
VEGF: vascular endothelial growth factor
SUCCESS-C: Simultaneous study of docetaxel-gemcitabine combination adjuvant treatment, and extended bisphosphonate and surveillance
WC: waist circumference
