# Erratum to: Feasibility outcomes of a presurgical randomized controlled trial exploring the impact of caloric restriction and increased physical activity versus a wait-list control on tumor characteristics and circulating biomarkers in men electing prostatectomy for prostate cancer

**DOI:** 10.1186/s12885-016-3025-3

**Published:** 2017-01-23

**Authors:** Wendy Demark-Wahnefried, Jeffery W. Nix, Gary R. Hunter, Soroush Rais-Bahrami, Renee A. Desmond, Balu Chacko, Casey D. Morrow, Maria Azrad, Andrew D. Frugé, Yuko Tsuruta, Travis Ptacek, Scott A. Tully, Roanne Segal, William E. Grizzle

**Affiliations:** 1 Department of Nutrition Sciences, University of Alabama at Birmingham (UAB), 346 Webb Nutrition Sciences Bldg., 1675 University Blvd, Birmingham, AL USA; 2 Department of Urology, UAB, Birmingham, AL USA; 3 Department of Human Studies, UAB, Birmingham, AL USA; 4 Department of Preventive Medicine, UAB, Birmingham, AL USA; 5 Department of Molecular & Cellular Pathology, UAB, Birmingham, AL USA; 6 Department of Cell, Developmental & Integrative Biology, UAB, Birmingham, AL USA; 7 Department of Microbiology, UAB, Birmingham, AL USA; 8 Urology Centers of Alabama, Birmingham, AL USA; 9 Department of Medicine, University of Ottawa, Ottawa, Ontario Canada; 10 Department of Pathology, UAB, Birmingham, AL USA

## Erratum

In this version of this article that was originally published [1] there was an error in the figure “CONSORT diagram (Fig. 3)”. The labels of the Experimental (Weight Loss) Intervention and the Wait–List Control are currently incorrect and should be exchanged for one another.

The corrected diagram is shown below:

**Fig. 3:**
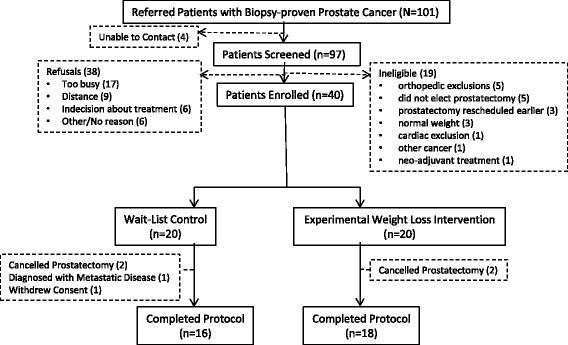
CONSORT diagram
